# Clinical performance of an analytically validated assay in comparison to microarray technology to assess PITX2 DNA-methylation in breast cancer

**DOI:** 10.1038/s41598-018-34919-1

**Published:** 2018-11-15

**Authors:** Gabriele Schricker, Rudolf Napieralski, Aurelia Noske, Elodie Piednoir, Olivia Manner, Elisabeth Schüren, Jürgen Lauber, Jonathan Perkins, Viktor Magdolen, Manfred Schmitt, Kurt Ulm, Wilko Weichert, Marion Kiechle, John W. M. Martens, Olaf G. Wilhelm

**Affiliations:** 1 Therawis Diagnostics GmbH, Grillparzerstrasse 14, 81675 Munich, Germany; 2 Institute of Pathology, Klinikum rechts der Isar, Technische Universität München, Ismaninger Strasse 22, 81675 Munich, Germany; 3 HalioDx Luminy Biotech Entreprises, 163 Avenue de Luminy, 13009 Marseille, France; 4 QIAGEN GmbH, Qiagen Strasse 1, 40724 Hilden, Germany; 5 QIAGEN Manchester Ltd., Lloyd Street North, Manchester, M15 6SH United Kingdom; 6 Department of Obstetrics and Gynecology, Clinical Research Unit, Klinikum rechts der Isar, Technische Universität München, Ismaningerstrasse 22, 81675 Munich, Germany; 7 Institute of Medical Informatics, Statistics and Epidemiology, Grillparzerstrasse 18, 81675 Munich, Germany; 8 Department of Medical Oncology and Cancer Genomics Netherlands, Erasmus MC, Wytemaweg 80, 3015 CN, Rotterdam, The Netherlands

## Abstract

Significant evidence has accumulated that DNA-methylation of the paired-like homeodomain transcription factor 2 (*PITX2*) gene can serve as a prognostic and predictive biomarker in breast cancer. PITX2 DNA-methylation data have been obtained so far from microarray and polymerase chain reaction (PCR)-based research tests. The availability of an analytically validated *in vitro* methylation-specific real-time PCR assay format (*therascreen* PITX2 RGQ PCR assay) intended for the determination of the percent methylation ratio (PMR) in the (PITX2) promoter 2 prompted us to investigate whether the clinical performance of these different assay systems generate comparable clinical outcome data. Mathematically converted microarray data of a previous breast cancer study (n = 204) into PMR values leads to a PITX2 cut-off value at PMR 14.73. Recalculation of the data to experimentally equivalent PMRs with the PCR PITX2 assay leads to a cut-off value at PMR 12 with the highest statistical significance. This cut-off predicts outcome of high-risk breast cancer patients to adjuvant anthracycline-based chemotherapy (n = 204; Hazard Ratio 2.48; p < 0.001) comparable to microarray generated results (n = 204; Hazard ratio 2.32; p < 0.0001). The *therascreen* PITX2 RGQ PCR assay is an analytically validated test with high reliability and robustness and predicts outcome of high-risk breast cancer patients to anthracycline-based chemotherapy.

## Introduction

Breast cancer is the most common malignancy in women with >464,000 new cases diagnosed in 2012 in Europe^1^. The St. Gallen classification includes steroid hormone receptor status and human epithelial growth factor receptor 2 (HER2) status, nodal status, tumor size and other clinicopathological factors to classify low- (10%), medium- (65%) and high-risk (25%) patient groups and help to guide therapy decision^2,3^. These prognostic factors provide information about the future clinical course of the disease e.g. disease-free survival (DFS) and overall survival (OS) of patients not subjected to any systemic cancer therapy. Different from that, predictive factors assess the probability of a cancer patient to respond and to benefit to a specific anti-cancer therapy. Those predictive biomarkers (proteins) are for example estrogen receptor (ER) and progesterone receptor (PR), the HER2 (human epithelial growth factor receptor 2) receptor, and the urokinase-type plasminogen activator (uPA) and its inhibitor PAI-1 (ASCO - American Society of Clinical Oncology: http://asco.org/practice-guidelines). ER and PR receptor status are usually assayed by immunohistochemistry (IHC), HER2 by IHC or by Fluorescence *In-Situ* Hybridization (FISH) or uPA/PAI by enzyme linked immunosorbent assay (ELISA).

Tumors classified as high-risk cancer with poor prognosis include triple-negative and HER2 positive breast cancer, patients with lymph node involvement^4^ and patients classified through multigene mRNA signatures such as Endopredict® (Myriad Genetics Inc., Salt Lake City, Utah, USA), OncotypeDX® (Genomic Health, Redwood City, CA, USA) or Mammaprint® (Agendia Inc., Irvine, CA, USA) to be at high risk to develop metastasis^5,6^. Standard of care treatment of those high-risk breast cancer patients is an anthracycline-based chemotherapy. In addition, an adjuvant endocrine therapy will be administered if tumor cells are ER and/or PR positive. However, not all patients respond to anthracycline-based chemotherapy, and part of the patients suffer from side-effects without any benefit. Until now biomarkers to predict outcome to anthracycline-based chemotherapy are of high unmet medical need.

DNA-methylation of the PITX2 (paired-like homeodomain transcription factor 2) gene as a predictive and prognostic biomarker for patient selection has received increasing attention not only in breast cancer^7–9^. PITX2 is a transcription factor, which is involved in pituitary-specific gene regulation and left-right patterning during embryonic and organogenic development^9^. It has been shown that DNA-methylation of the PITX2 promoter gene predicts risk of distant metastasis in node-negative, hormone receptor-positive breast cancer^10–12^. Significant evidence has accumulated that PITX2 methylation predicts outcome in lymph node-positive, ER positive, HER2 negative breast cancer patients to adjuvant anthracycline-based chemotherapy and, thus, will support the clinicians to the most effective therapy option^13,14^. In these studies, different technological platforms for DNA-methylation analysis were used and performed on different types of tissue specimens. Those studies include a proprietary microarray platform developed by the company Epigenomics AG (Berlin, Germany) for on DNA methylation based biomarker screening using bisulfite converted DNA (bisDNA) specific probes for the methylated and unmethylated status of CpG residues^11,13^ or a ‘research use only’ (RUO) real-time PCR assay covering three of the most relevant CpG residues for clinical response prediction in tamoxifen treated breast cancer patients^11^.

Recently, we developed an analytically validated PITX2 DNA-methylation assay (*therascreen* PITX2 RGQ PCR^15^, which is by now CE-marked and commercially available^9^. Here we describe results of the technical evaluation study of the *therascreen* PITX2 RGQ PCR assay according to Clinical and Laboratory Standards Institute (Clinical and Laboratory Standards Institute - CLSI; https://clsi.org/) guidelines for lymph node positive, ER positive, HER2 negative breast cancers. The *therascreen* PITX2 assay is deployable for routine diagnostic setting and the determined PMR value will aid clinicians to identify patients who are more versus less likely to benefit from adjuvant anthracycline-based chemotherapy. The goal of the present study was to investigate whether the clinical relevance of PITX2 DNA-methylation determined by the novel *therascreen* PITX2 DNA-methylation assay delivers the same results compared to the previously applied microarray technology^13^ and research PCR assay^11^.

## Material and Methods

### Sample preparation, DNA extraction and bisulfite conversion

Sample preparation from Fresh-Frozen (FF) tumor tissue: after surgical removal, breast tumor tissues were placed immediately on ice until examination by the pathologist. Thereafter, tissue was snap-frozen and stored in liquid nitrogen until further use. FF tumor tissue was processed to tumor cell nuclei pelleted at 100,000 × g at Erasmus Medical Center, Rotterdam as described^13,16^. Samples were transferred to Therawis Diagnostics GmbH, Munich. Genomic DNA (gDNA) was extracted from 10–30 mg FF tumor cell nuclei pellets using the QIAamp^®^ DNA Mini Kit (QIAGEN, Hilden, Germany; Catalog No.: 51304/51306).

Formalin-fixed paraffin-embedded (FFPE) tumor tissue samples were prepared by fixation for 12–24 hrs in 10% neutral buffered formalin and embedded in paraffin. Tissue blocks were stored at room temperature and archived at the Institute of Pathology, Klinikum rechts der Isar, Technische Universität München until further use. gDNA was purified from one to two 5 µm FFPE tissue sections with a total surface area ≥100 mm^2^ using the QIAamp DNA DSP FFPE kit (Qiagen, Hilden, Germany; Catalog No.: 60404).

DNA quantification was performed with the QIAxpert spectrophotometer (Qiagen, Hilden, Germany) using the QIAamp DNA plugin with internal blank calibration for elution buffer ATE with the OD_260_ readout of total nucleic acids. DNA bisulfite conversion of the gDNA was performed using the Epitect Fast DNA Bisulfite Kit (Qiagen, Hilden, Germany, Catalog No.: 59824/59826) using 200–1000 ng input into the bisulfite conversion according to the *therascreen* PITX2 RGQ PCR Kit workflow^15^. This equals a bisDNA input in the qPCR into quantitative PCR (qPCR) of 53–267 ng bisDNA/well. All samples are measured in duplicates.

### Methylation-specific custom microarray

**Figure 1:**
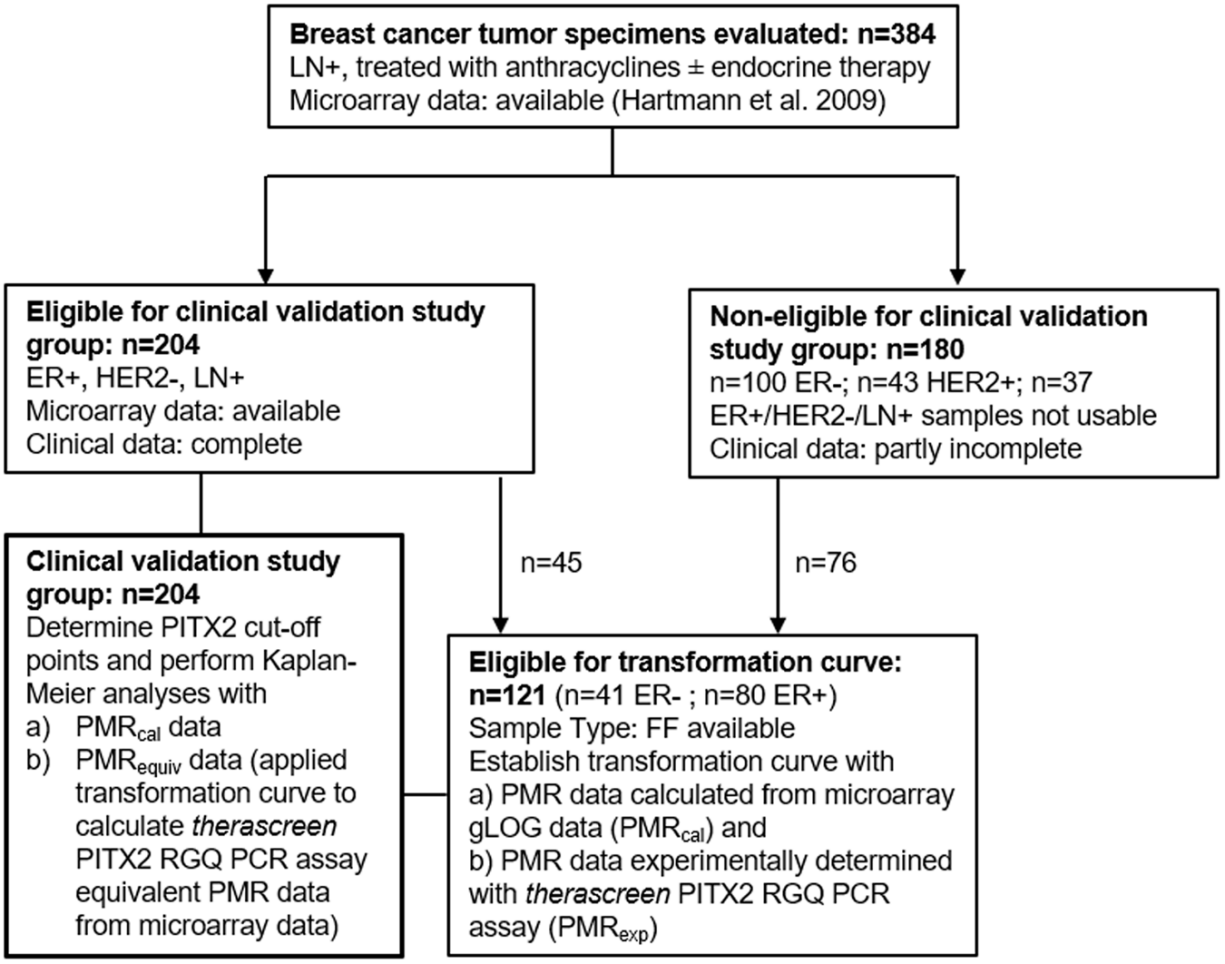
Consort diagram with samples for clinical evaluation of *therascreen* PITX2 RGQ PCR assay. ER - Estrogen receptor; HER2 - human epithelial growth factor receptor 2; LN - lymph node; FF - fresh-frozen.

A customized methylation-specific oligonucleotide array was developed by Epigenomics AG for 61 genes for initial screening in a population of 384 breast cancer samples^13^. CpG sites from regulatory regions of the candidate genes were amplified in multiplex PCR reactions labeled with the fluorochrome Cy5 from bisulfite-treated genomic DNA with bisulfite-converted DNA specific primer pairs. In total, 64 PCR amplificants representing 61 genes were pooled and hybridized to the microarray on which detection oligonucleotides for methylated (CG) and nonmethylated (TG) gene copies were spotted. This allowed for simultaneous quantitative measurement of unmethylated and methylated copies of the genes. Microarrays included 4 oligonucleotide pairs for each of the 64 PCR amplificates (total of 256 pairs). Each probe pair covered between one and three CpG dinucleotides in the regulatory regions of the respective candidate gene. The methylation score for each CpG site was calculated from the fluorescence intensity values of the methylated (=FIm) and unmethylated (=FIu) oligonucleotides. To stabilize the variance, the score was transformed using the generalized log transformation (gLOG): methylation score = gLOG(FIm/FIu)^17^. For statistical analysis, methylation scores for each amplificate were determined by averaging measurements from all probe pairs belonging to one amplificate using the median. Multiple amplificates from the same candidate gene entered data analysis independently. According to Maier *et al*. (shown in Fig. 1) 3 CpGs with the most relevant clinical impact were selected from the PITX2 promoter 2 gene (PITX2P2; 4q25) region on the respective custom array, which were represented by two of the four probe pairs^11^. These 3 CpGs are covered by the Taqman probes of the *therascreen* PITX2 RGQ assay.

### *therascreen* PITX2 RGQ PCR assay

The *therascreen* PITX2 RGQ PCR Kit (QIAGEN, Catalog no. 873211) is a real-time DNA-methylation-specific PCR-based assay (qMSP) and exploits the quantitative PCR (qPCR) oligonucleotide hydrolysis principle of two TaqMan probes (labelled with fluorescent dye FAM^TM^ for fully methylated and HEX^TM^ for fully unmethylated probe) in combination with methylation-unspecific primers. The sample type is bisDNA, i.e., bisulfite converted genomic DNA (gDNA). This assay uses one pair of primers amplifying all bisulfite-converted target sequences of the PITX2 gene promoter 2 by its methylation specific probes^15^. PITX2-probe and primer system specifications: Entrez gene ID: 5308. Amplicon length 144. Reference sequence (Ref Seq) ID: NT_016354.18 (4q25. Detected CpG in Ref Seq: 3 CpG in 36106573–36106600^11^. After bisulfite exposure to distinguish between methylated and unmethylated PITX2, the percent methylation ration (PMR) of three CpG motifs of the PITX2 gene promoter 2 is quantified by qMSP and calculated by the Rotor-Gene AssayManager^®^ software with Gamma Plug-in plus a kit-specific PITX2 Assay Profile for automated analysis and quality control including all validity criteria. Validity criteria for run controls and sample testing include cycle threshold (Ct), amplification curve anomalies, PMR range for the controls (PITX2 RGQ PCR reference 50, PITX2 RGQ PCR reference low, PITX2 RGQ PCR negative control (NC), PITX2 RGQ PCR NTC (NTC)) and delta-PMR threshold for sample duplicates as described in detail in the handbook^18^.

The PMR (percent methylation ratio) is calculated applying the following formula: PMR = 100/(1 + 2exp(Ct_FAM(methylated)_ − Ct_HEX(unmethylated)_)]^15^. The PITX2 qMSP assay was optimized for the usage as an *in vitro* diagnostic assay following the CLSI guidelines (https://clsi.org/; see also^18^). PCR analysis is performed on Rotor-Gene Q MDx 5plex HRM instrument (Qiagen, Hilden, Germany).

### Patients and tumor samples

For the analytical and technical evaluation of the *therascreen* PITX2 RGQ PCR assay, Formalin-Fixed Paraffin-Embedded (FFPE) tumor tissue samples from invasive breast cancers (n = 131; thereof 61 ER+ and 68 ER−) were collected at the Institute of Pathology, Technische Universität München. From these cases incomplete clinical and follow-up data were available. Therefore, the FFPE samples were only used to optimize the workflow, perform the analytical and technical evaluation including determination of DNA extraction yields, pass rates (run pass rate and sample pass rate), and tumor heterogeneity.

For the establishment of the transformation curve with the *therascreen* PITX2 RGQ PCR assay, Fresh frozen (FF) tumor tissue samples (n = 121) processed to tumor cell nuclei pellets were provided by the Erasmus Medical Center (EMC), Rotterdam, The Netherlands.

From a study published by Hartmann *et al*. 2009, 384 patients were evaluated according to the following eligibility criteria: invasive breast cancer, tumor stage pT1 to pT3, histologically confirmed lymph node involvement (>pN1), availability of clinical follow-up data of at least 5 years, availability of PITX2 methylation-specific microarray data, and, finally, standard adjuvant anthracycline-based chemotherapy (no dose-dense therapy, no other primary systemic chemotherapy, except hormonal therapy, and no additional taxanes) (Fig. 1).

From 121 of the 384 patients (Fig. 1), both fresh-frozen tumor tissue specimens and microarray data of PITX2 DNA methylation were available, but not all samples met the inclusion criteria of the clinical study population Also, the available clinical data of this group of 121 patients were partly incomplete and, therefore, could not be used for outcome analysis. The 121 samples, which were collected at the Erasmus Medical Center in Rotterdam were used to analyze the correlation between mathematically converted microarray gLOG (FIm/FIu) data to PMR values [PMR_calc_ = 2exp(gLOG (FIm/FIu))] and their respective PMR values experimentally (PMR_exp_) determined with the *therascreen* PITX2 RGQ PCR assay according to the formula: PMR_exp_ = 100/[1 + 2exp(Ct_FAM(methylated)_ − Ct_HEX(unmethylated)_)] from fresh frozen tumor tissue samples.

204 patients (ER positive, HER2 negative) of the 384 patients were selected for the clinical validation study group (Fig. 1). This clinical validation study group was considered for determination of PITX2 cut-off points and to perform Kaplan-Maier analyses by using available microarray gLOG (Flm/Flu) data from fresh-frozen tissue samples transformed into PMR values by a) mathematically calculation (PMR_calc_) and b) applying the established transformation curve to calculate *therascreen* PITX2 RGQ PCR assay equivalent PMR data (PMR_equiv_) and using the clinical data of this group (Fig. 1 – bold box). The histopathological and clinical criteria of this group are summarized in Table 1.

**Table 1:** Clinical Study Population.

Clinical characteristics	n (%)
**No. cases**	204 (100)
**Age at time of diagnosis**	
<50 years	90 (44)
≥50 years	114 (56)
**Tumor stage (pT)**	
T1	74 (36)
T2, T3, T4	128 (114; 14; 0) (63)
TX (unknown)	2 (1)
**Involved lymph nodes**	
1–3	110 (54)
>3	94 (46)
**ER status**	
positive	204 (100)
**HER2/neu status**	
negative	204 (100)
**Therapy**	
Anthracycline-based plus endocrine therapy	96 (47)
Anthracycline-based without endocrine therapy*	108 (53)
**Disease recurrence**	
Yes	94 (46)
No	110 (54)
**Overall survival (event)**	
Yes	46 (23)
No	158 (77)

Clinical and histopathological characteristics of patients (n = 204). *With the ASCO/CAP (American College of Pathology) recommendation in 2010, a consensus threshold reporting ER as positive was set at 1%^32^. Consequently, prior to the change of guidelines, patients with low level ER expression (between 1% and 10%) were not necessarily treated with endocrine therapy.

### Experimental studies involving human tissue material

Any experiments on human tissue material cited which were conducted in cooperation with the Department of Obstetrics and Gynecology (Frauenklinik) and the Institute of Pathology of the Technische Universität München, Munich, Germany, and with Erasmus Medical Center, Rotterdam, The Netherlands, were done in accordance with the Declaration of Helsinki (1964) and in accordance to the Code of Conduct of the Federation of Medical Scientific Societies in the Netherlands (www.federa.org/codes-conduct). The laboratory experiments were performed with the human subjects’ understanding, who provided written informed consent for using the respective tissue specimens. The present study was approved by the local Ethical Committee of the Technische Universität München – Faculty of Medicine, Munich, Germany.

### Statistical methods

Fractional polynomial approach was used to analyze the correlation between PMR data calculated from gLOG values of methylation-specific microarray data (PMR_calc_) and PMR data measured with the *therascreen* PITX2 RGQ assay (PMR_exp_) values to determine the best-fitting function^19^. gLog(2) fluorescence intensity values (gLOG Flm/Flu)) of PITX2 on a methylation specific oligonucleotide microarray^20^ were transformed into PMR values (PMR_calc_) by the formula: PMR_calc_ = 2exp(gLOG) to allow for comparability with PMR data (PMR_exp_) from the *therascreen* PITX2 RGQ PCR assay according to the respective probe CT read-outs [PMR_exp_ = 100/(1 + 2exp(Ct_FAM(methylated)_ − Ct_HEX(unmethylated)_))]. The resulting polynomial regression analysis function [PMR_equiv_ = 12.12773 + 11.61198*((2exp(gLOG)/10*100) − 1.491) − 0.0938867*((2exp(gLOG)/10*100)^3^ − 3.314))] was used to mathematically calculate PMR_calc_ values into *therascreen* PITX2 RGQ assay equivalent PMR values (PMR_equiv)_ sample by sample from the Clinical Study Population (n = 204; Fig. 1).

Disease-free survival (DFS) was the primary endpoint and defined as the time from primary surgery to the first documented event, which includes any of the following: local recurrence of disease (n = 19) or distant metastasis (n = 70) or contralateral breast cancer (n = 5). Analysis was performed for DFS follow-up time of 120 months. The PITX2 cut-off value for disease-free survival (DFS) was established with the “maximum-selected log-rank statistics” using the maxstat.test function as implemented by the program library “maxstat” of the program “R” (R Development Core Team 2012)^21^. The PITX2 cut-off value is the Percentage Methylation Ratio (PMR) of PITX2 which separates good survivors and poor survivors (responder vs. non-responder) by using the log-rank statistics algorithm (R Software version: R version 3.4.1, 2017; the R Foundation for Statistical Computing).

## Results

### Assay performance

The verification data and the analytical validity of the therascreen PITX2 RGQ PCR DNA-methylation assay including limit of blank (PMR 0 for the methylated probe; PMR 98 for the unmethylated probe), limit of detection (PMR 4 for the lower limit; PMR 92 for the upper limit), repeatability and reproducibility was tested with three biological samples (PMRs 7, 16, and 77) in a single-site precision study (variability for each sample = 20.39%, 21.76%, 4.23%, respectively; average of 15.46%) and multi-site precision study (variability for each sample = 16.93%, 28.72%, 4.61%, respectively; average of 16.75%) according to CLSI/NCCLS/EP5-A3 guideline^22^, including intra-run variability (for each sample = 12.29%, 19.99%, 3.90%, respectively; average of 12.06%) and inter-run variability (for each sample = 13.90%, 28.72%, 4.25%, respectively; average of 15.62%)^15,18^. Furthermore, interfering substances (8 substances; no biological impact on PMR results) according to CLSI/NCCLS/EP7-A2 guideline^23^, cross-contamination (1.3%), and in-use timeframe (within 24 hours) were tested and described in detail^15,18^. The results showed that the therascreen PITX2 RGQ PCR DNA-methylation assay is reliable, robust and ready-to-use for routine diagnostics.

### DNA yield from FFPE tumor sections

To verify the successful application of the *therascreen* workflow from FFPE material to PCR-based results, we used 131 FFPE breast cancer samples (63 estrogen receptor positive and 68 estrogen receptor negative), which were collected at the Institute of Pathology, Technische Universität München, as an independent cohort. From those samples, the genomic DNA (gDNA) was extracted from one to two 5 µm FFPE-tumor tissue sections with a total surface area ≥100 mm^2^. DNA quantification was performed with the QIAxpert spectrophotometer (Qiagen, Hilden, Germany) using the QIAamp DNA plugin with internal blank calibration for elution buffer ATE with the OD_260_ readout of total nucleic acids. For 110 tumor samples more than 400 ng of gDNA were obtained per sample, indicating an overall gDNA extraction pass rate of 84% for subsequent bisulfite conversion at the recommended gDNA input according to the assay format of 400 ng input (Table 2). The gDNA yield for the minimal gDNA input of 200 ng was achieved in 125 samples (95.4%). Samples with a tissue area below 100 mm^2^ (n = 5) had yields between 85 and 440 ng. In those cases, several pooled 5 µm sections for gDNA extraction were required.

**Table 2:** Genomic DNA (gDNA) extraction yields. Amount of gDNA from one 5 µm section and the respective tissue section areas. Total numbers of samples: 131. The input of 200–1000 ng gDNA into bisulfite conversion equals input of 53–267 ng bisDNA per well in the qPCR.

gDNA yield	Samples	
[ng]	n (out of 131)	[%]
<200	6	4.6
≥200	125	95.4
≥400	110	84.0
≥1000	79	60.3

### Pass rates for qPCR runs and samples (DNA input)

Six runs were performed to analyze 104 FFPE tissue tumor samples. The assays were performed with the recommended DNA input of 400 ng for bisulfite conversion (n = 104) resulting in a final input of 106 ng bisulfite-converted DNA per well into qPCR. The six assay runs performed were all valid (Table 3) according to the PITX2 Assay Profile for the *therascreen* PITX2 RGQ PCR Kit (see Materials and Methods).

**Table 3:** Pass rates for qPCR runs and samples (DNA input).

	Total (n)	Valid results	Invalid results	Rate [%]
Run pass rate - qPCR run performed	6	6	0	100
Sample pass rate - first-time	104	91	13	87.5
Sample pass rate - re-test	104	99	5	95.2

All criteria for run validity and sample DNA input validity were automatically applied according to the PITX2 Assay Profile of the *therascreen* PITX2 RGQ PCR Kit^18^. All determinations were performed in duplicates.

The first-time sample pass rate was 87.5%. If invalid test results were obtained by the automated analysis software of the PITX2 Assay Profile (see Materials and Methods), the qPCR run was repeated. According to the retest workflow of the *therascreen* PITX2 RGQ PCR Kit, a minimum of 200 ng gDNA up to 1000 ng gDNA input was used for bisulfite conversion. Upon retesting of 13 samples, the overall sample pass rate of valid results increased to 95.2%.

### Tumor heterogeneity

To investigate the heterogeneity of PITX2 DNA-methylation within the same tumor sample, 5 consecutive tissue sections of the same tumor were analyzed. As denoted in Table 4, six analyzed tumor specimens gave coefficients of variations (CV) from 7.59% to 29.70%. However, the intra- and inter-assay variability range of the *therascreen* assay format is between 3.90–28.72%^18^. The variability range observed in consecutive tumor sections with 7.59% to 29.70% is in the same range. Therefore, the contribution of tumor heterogeneity to PITX2 PMR value variability seems to be rather low.

**Table 4:** Tumor heterogeneity of PITX2 DNA-methylation.

Tumor Sample	Mean Value PMR	CV PMR	CV (%)	PMR range (absolute)
Tu 16.1–5	11.36	3.05	26.84	5.69–16.11
Tu 10.1–5	21.53	5.99	27.81	12.79–31.21
Tu 12.1–5	30.13	8.95	29.70	14.50–46.02
Tu 14.1–5	57.86	12.47	21.56	41.76–80.55
Tu 15.1–5	73.70	6.87	9.32	64.00–83.60
Tu 03.1–5	54.82	4.16	7.59	46.89–62.55

Five consecutive tissue sections (Tu xx.1–5) of six tumor specimens Tu 16, 10, 12, 14, 15 and 3 were analyzed in duplicates for each tissue section and the mean PMR value, the coefficients of variation (CV) for PMR and in percent were determined. The PMR range represents the CV in absolute PMR values.

### Comparison of cut-off and clinical data derived from microarray analysis and therascreen PITX2 RGQ PCR assay format

Hartmann *et al*. 2009 analyzed the performance of the PITX2 marker in 241 ER-positive, HER2-negative, and lymph-node positive tumor tissue samples showing that PITX2 promoter 2 (PITX2P2) hypermethylation was associated with a high risk of distant recurrence (time to distant metastasis) in the patient cohort (HR = 1.66; p = 0.002) and poor DFS (HR = 1.47; p = 0.0084) with the amplificate designed for the promoter of transcripts A and B of PITX2 (PITX2P2). The hazard ratio (HR) in this study was calculated relative to an increment from the lower quartile to the upper quartile^13^.

**Figure 2:**
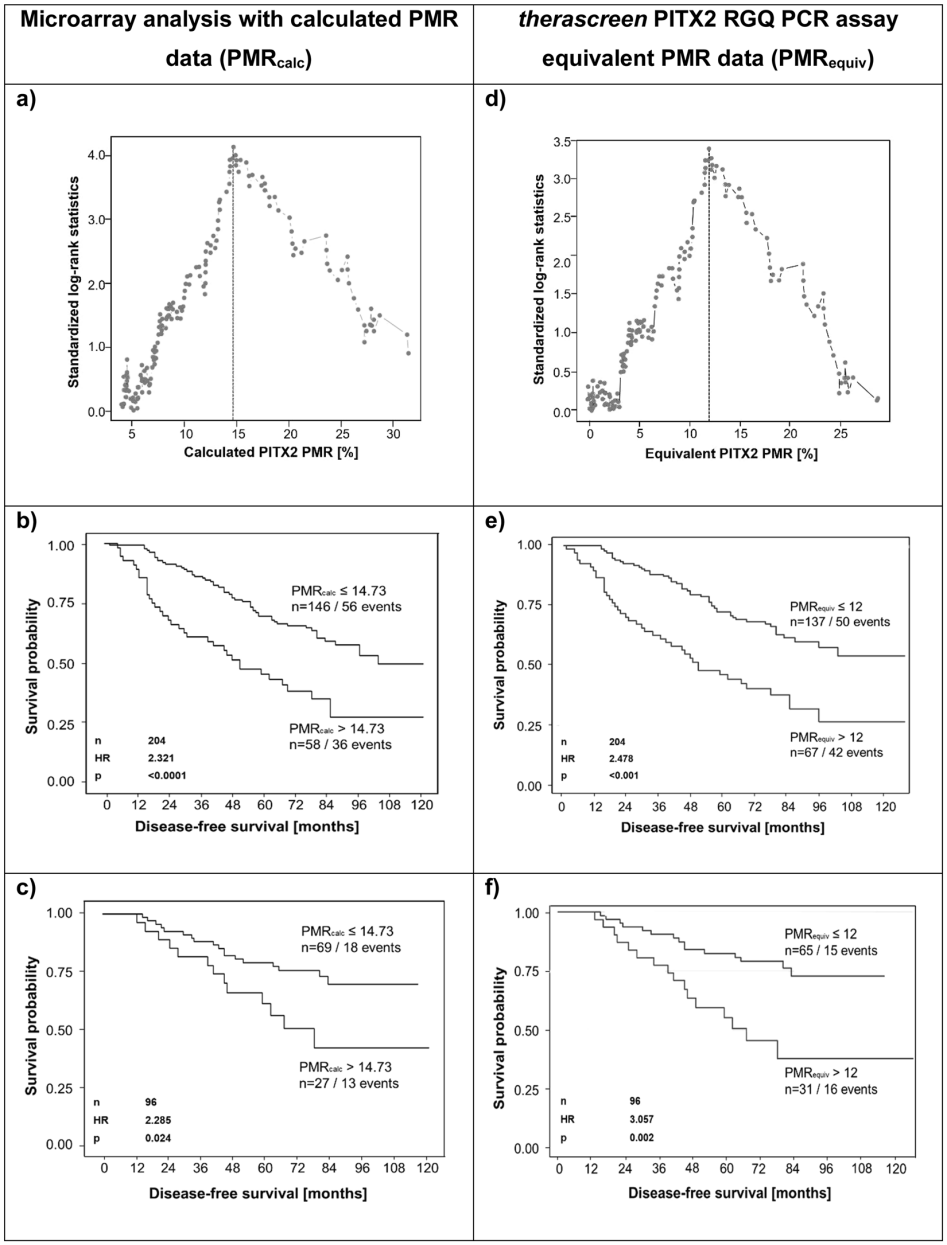
Determination of PITX2 cut-off points and Kaplan Meier analysis. (**a)** Statistical analysis of PITX2 cut-off points (PMR) for all patients (n = 204). Microarray gLOG data were converted with 2exp(gLOG) function to calculated PITX2 PMR data (PMR_calc_); x-axis: Calculated PITX2 PMR data [%]; y-axis: Standardized log-rank statistics. Dashed vertical line marks the maximum cut-off point of PITX2 PMR_calc_ at 14.73. (**b)** Kaplan-Meier analysis for DFS of high-risk breast cancer patients (n = 204) using the maximum PITX2 cut-off point (PMR_calc_ = 14.73). Patients were treated with anthracycline-based chemotherapy ± endocrine therapy (HR 2.321; p < 0.001). Upper line: patients with PMR ≤ 14.73. Lower line: patients with PMR > 14.73. (**c)** Kaplan-Meier analysis for DFS of subgroup of high-risk breast cancer patients treated with anthracycline-based chemotherapy and additional endocrine therapy (n = 96) using the maximum PITX2 cut-off point (PMR_calc_ = 14.73) (HR 2.285; p = 0.024). Upper line: patients with PMR ≤ 14.73. Lower line: patients with PMR > 14.73. (**d)** Statistical evaluation of PITX2 DNA percent methylation ratio (PMR) cut-off points. PMR data (n = 204) are equivalent (PMR_equiv_) to *therascreen* PITX2 RGQ PCR assay determined PMR values. x-axis: equivalent PITX2 PMR [%]; y-axis: Standardized log-rank statistics. The dashed vertical line marks the maximum log-rank statistic at a PITX2 PMR cut-off point of 12. (**e)** Kaplan-Meier analysis for DFS of high-risk breast cancer patients (n = 204) treated with anthracycline-based chemotherapy ± endocrine therapy using the optimized PITX2 cut-off point of PMR_equiv_ = 12 (HR 2.478; p < 0.001). Upper line: PMR ≤ 12. Lower line: PMR > 12. (**f)** Kaplan-Meier analysis for DFS of high-risk breast cancer patients treated with anthracycline-based chemotherapy plus endocrine therapy (n = 96) using the optimized PITX2 cut-off point of PMR_equiv_ = 12 (HR 3.057; p = 0.002). Upper line: PMR ≤ 12. Lower line: PMR > 12. Kaplan-Meier analysis: n = 2 patients with DFS at 147 and 166 months were censored for DFS analysis with follow-up time of 120 months.

In order to examine if the clinical relevance (hazard ratio) are similar with data generated by microarray analysis or compared to data generated with the *therascreen* PITX2 assay for DFS the following strategy was pursued: 204 of 241 patient data were eligible (Fig. 1) and used for our analyses. The microarray data (gLOG data; Hartmann *et al*. 2009) from fresh frozen (FF) tissue of high-risk breast cancer patients (Fig. 1; n = 204) were converted by mathematical calculation with 2exp(gLOG) function into PITX2 PMR_calc_ data and used to determine the best PITX2 cut-off value (PMR_calc_) for DFS as shown in Fig. 2a. A pronounced single peak is shown reaching the highest statistical difference (z = 4.15) of two groups of patients with long and short DFS at a cut-off value of PMR_calc_ 14.73. Kaplan-Meier analysis applying the cut-off value of PMR_calc_ 14.73 identifies patients who benefit from anthracycline-based chemotherapy (n = 204; ±endocrine treatment) with a significant longer DFS (HR 2.32, p < 0.0001) and those with less benefit from anthracycline-based chemotherapy (Fig. 2b).

**Figure 3:**
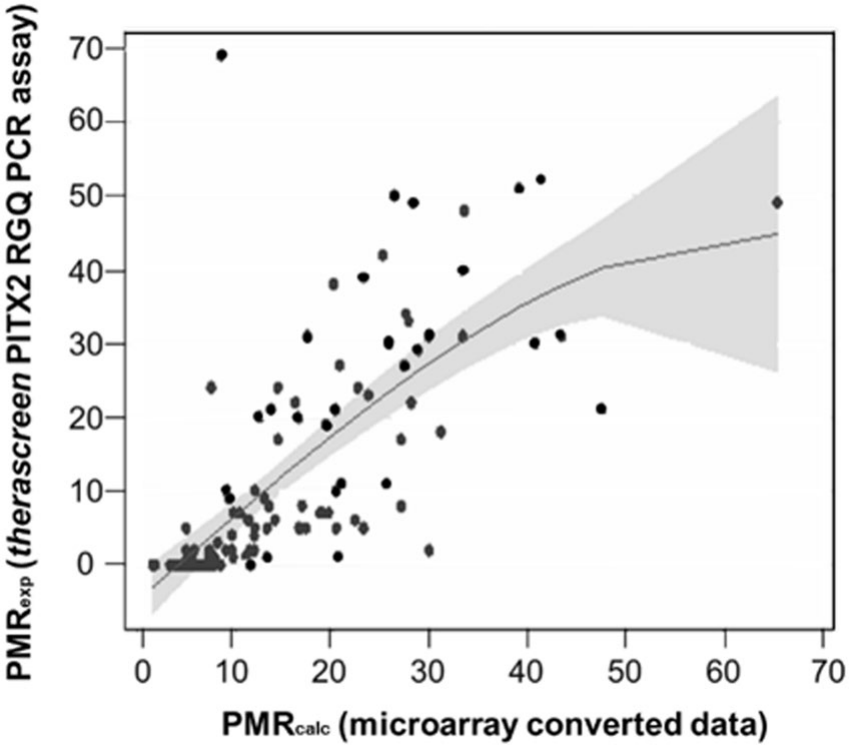
Establishment of the transformation curve. Correlation of the PMR_calc_ (mathematically converted microarray data) and PMR_exp_ (data determined by *therascreen* PITX2 RGQ PCR assay) with 121 samples (41 ER−; 80 ER+). Coefficient factors: r-squared = 0.5219; r = 0.722.

PITX2 DNA-methylation also identifies patients in the group of breast cancer patients who received in addition to anthracycline-based chemotherapy endocrine therapy (n = 96) who have a significant longer DFS and those patients who have a significantly shorter DFS (HR 2.285, p = 0.024) (Fig. 2c). In order to confirm these results with a PCR-based analysis method we determined the PITX2 DNA-methylation applying the analytically validated *therascreen* PITX2 assay in 121 tumor cell nuclei pellets from fresh frozen (FF) tissue samples available from the same study (Fig. 1). However, as the clinical data for these 121 samples were partly incomplete, we established a transformation curve with the PMR values derived from microarray (PMR_calc_) and qPCR (PMR_exp_; assessed with the *therascreen* PITX2 RGQ PCR Kit) from these 121 samples. This resulted in a moderate correlation coefficient factor of r = 0.722 (r-squared = 0.5219; n = 121) as shown in Fig. 3. The relative high number of zero values is due to the limit of detection of *therascreen* PITX2 RGQ PCR test which results in zero values for all PMRs between 0 and 4. The best fitting regression analysis function was determined by using the fractional polynomial approach (see statistical methods) and used to calculate PMR_calc_ values into *therascreen* PITX2 RGQ assay equivalent PMR values (PMR_equiv)_ sample by sample from the Clinical Study Population (n = 204; Fig. 1).

The resulting *therascreen* PITX2 RGQ PCR assay equivalent PMR data (PMR_equiv_) were used to determine the best PITX2 cut-off value leading to PMR_equiv_ = 12 for DFS as shown in Fig. 2d. The Kaplan-Meier curves with the applied cut-off value of PMR_equiv_ = 12 identifies patients with improved survival after anthracycline-based chemotherapy (n = 204; ±endocrine treatment; HR 2.478; p < 0.001) (Fig. 2e) or anthracycline-based chemotherapy plus endocrine therapy (n = 96; HR 3.057; p = 0.002) (Fig. 2f).

The observed hazard ratios applying the cut-off derived from converted microarray data with PMR_calc_ = 14.73 and from *therascreen* PITX2 RGQ PCR assay equivalent PMR data with PMR_equiv_ = 12 are quite comparable and 9 of 204 patients (4.4%) of the clinical validation group switch from the low-risk group into the high-risk group with applying PMR_equiv_ = 12. In current standard-of-care, this would lead to treatment of these patients with anthracycline-based chemotherapy.

## Discussion

The clinical relevance of the PITX2 DNA-methylation status in breast cancer has been described in several studies^8,10–14,24^. The precise role of PITX2 DNA-methylation in breast carcinogenesis remains not fully understood. One publication described a prognostic role of PITX2 DNA-methylation for the clinical course of breast cancer^10^. In the studies performed by Maier *et al*.^11^ and Harbeck *et al*.^11,12^, PITX2 DNA-methylation showed the strongest correlation with metastasis-free survival in node negative tamoxifen-treated breast cancer patients. The study of Maier *et al*., 2007 also showed good correlation of PITX2 DNA-methylation determined on a methylation specific oligonucleotide microarray and a research use only (RUO) qPCR assay^11^. These studies underline PITX2 DNA-methylation as a potential biomarker for predicting outcome in patients with node negative breast cancer.

The role of DNA-methylation in clinical cancer research gained significant attention. DNA-methylation analysis comprises single gene analysis on simple endpoint PCR based approaches, quantitative methylation specific PCR and oligonucleotide microarrays^25–27^. Evidence has been accumulated that DNA-methylation based markers - depending of the method applied - can be predictive or prognostic for clinical outcome and might improve diagnosis and treatment^28–31^. Hartmann *et al*., 2009 showed for the first time the clinical relevance of PITX2 methylation status in ER positive, HER2 negative, lymph node positive breast cancer patients treated with anthracycline-based chemotherapy^13^. PITX2 hypermethylation was associated with high risk of recurrence in that patient cohort [HR 1.66, p = 0.002] and was also associated with poor DFS (HR 1.47, p = 0.0084)^13^. However, the transformation of a research-based assay into a fully analytically validated test system rarely occurred and, therefore, only a few examples transitioned into clinical routine setting.

Array-based methodologies often are better suitable for marker discovery studies, whereas, single marker based quantitative DNA-methylation based assays are more reliable, cost-effective, sensitive and robust for high-throughput analysis in diagnostic or clinical chemistry units.

Therefore, a real-time PCR-based PITX2 marker assay as RUO test was developed for verification studies of the PITX2 marker^11,12^. Harbeck *et al*., 2008 showed that this RUO qPCR-based test can be reliably used in high-throughput studies (n = 399). This PCR-based assay demonstrated high reproducibility in replicate measurements (r ≥ 0.95, n = 150). Furthermore, Harbeck *et al*. (2008) showed, that PITX2 PMR values correlated between matched fresh frozen and FFPE tissue samples (n = 89, r = 0.81). Therefore, an expansion of the PITX2 test for assessment of more easily available FFPE material was indicated. The authors also concluded that tumor heterogeneity does not impact the analysis of PMR (percent methylation ratio) values in FFPE tissue blocks^12^.

The present technical evaluation study was based on the development of the aforementioned Research Use Only (RUO) assay into a CE certified *in vitro* diagnostics (IVD) kit including optimization of the workflow and analytical and technical verification and validation of the assay for its use in routine diagnostics. The assay was also optimized for use of the more easily available FFPE material. For the *therascreen* PITX2 RGQ assay single-site and multi-site precision studies were performed according to CLSI guideline^22^ for technical certification of the assay, proving high robustness (average coefficient of variation (CV) at about 16%). Because the average coefficient of variation was determined to 16%, there is a grey area in absolute PMR values around the cut-off of PMR 12 between PMR 10 to 14. For patients with tumor PMR results of PITX2 DNA-methylation in this grey area, the physician would very likely take additional clinical parameters for a therapy recommendation into account. The overall sample pass rate of DNA extraction for qPCR analysis was 95%, e.g. samples yielded sufficient DNA amounts for input into bisulfite conversion and overall 95% of all samples gave valid results according to the certified assay profile validity criteria^18^. These data emphasize that the *therascreen* PITX2 RGQ assay can reliably be used for analysis of FFPE-derived material. The amplifyable copy numbers assessed based on CT values of each sample did not correlate with the overall DNA yield, showing that DNA integrity in the original FFPE sample is critical on test outcome. Nevertheless, one 5 µm section with more than 100 mm^2^ tissue area is sufficient to achieve an overall success rate of more than 95% with the *therascreen* PITX2 qPCR workflow.

The present study confirms applying the analytically validated *therascreen* PITX2 RGQ PCR assay that PMR values derived from fresh-frozen tissue samples correlate (r = 0.722; r-squared = 0.5219; n = 121) with the microarray results from the Hartmann collective^13^. We could also demonstrate a statistically highly significant correlation between PITX2 DNA methylation *(therascreen* PITX2 RGQ PCR assay equivalent PMR data with PMR_equiv_ = 12) and clinical outcome (DFS) for patients treated with anthracycline-based chemotherapy.

Predictive tests to avoid undertreatment or overtreatment of high-risk breast cancer patients are urgently needed. Therefore, a test which can predict outcome in patients receiving anthracycline-based chemotherapy is of high unmet medical need because it would improve the compliance of patients, who will most likely benefit, to stay on therapy despite potential toxic side effects. Vice versa patients who will most likely not benefit might be considered for an alternative treatment option.

The *therascreen* PITX2 RGQ PCR kit is now commercially available, and it is intended to be used by qualified users trained in molecular biology techniques and in *in vitro* diagnostic procedures. The *therascreen* PITX2 RGQ PCR kit with the determined cut-off value of PMR 12 will allow identification of high-risk, lymph node-positive, estrogen-receptor positive, HER2-negative breast cancer patients treated with anthracycline-based chemotherapy with good versus poor outcome. Future studies using independent cohort of ER+ breast cancer FFPE tissue samples and also prospective clinical trials are required to further substantiate the clinical cut-off of PMR 12. In conclusion, PITX2 DNA-methylation determined with the analytically validated *therascreen* PITX2 RGQ PCR assay may improve personalized breast cancer management and treatment decision in high-risk (lymph node-positive, estrogen-receptor positive, HER2-negative) breast cancer.
